# The meaning of ‘risk’ in mental health care: a qualitative study of its usage in clinicians’ language

**DOI:** 10.1192/j.eurpsy.2025.1562

**Published:** 2025-08-26

**Authors:** H. Barker, L. Sambrook, P. Saini, C. Bu, R. Nathan

**Affiliations:** 1 School of Clinical Medicine, University of Cambridge, Cambridge; 2 Liverpool John Moores University, Liverpool; 3 CWP NHS Foundation Trust; 4 University of Chester, Chester, United Kingdom

## Abstract

**Introduction:**

Although there is good empirical data on factors that predict harmful outcomes, and standardised approaches to risk assessment have been developed, there remains a disconnect between the academic study of risk and routine clinical practice. This is exemplified by (i) the outstanding uncertainty about how to use predictive models for everyday clinical decision-making, and (ii) the use of predictive methodology to test tools that eschew prediction. The disconnect is, in part, a consequence of the varied use of the notion of ‘risk’ within and between academia and clinical practice.

**Objectives:**

To derive a more nuanced understanding of the meaning of ‘risk’ in clinical practice.

**Methods:**

After reading clinical vignettes, participants (all practising clinicians, n=18) took part in semi-structured interviews regarding clinical decision-making. The interview transcripts were subject to thematic analysis using a novel approach to the analysis of ideas in expressed language (in this case the idea of ‘risk’) which draws on philosophical and intellectual history methodologies (derived from the work of Wittgenstein, and Skinner respectively).

**Results:**

The use of risk by participants varied according to the extent and type of its spatial location (figure 1).

In many cases, ‘risk’ was used in a disembodied (i.e., dislocated) way (e.g., ‘what is the risk,’ ‘risk will increase’).

When locatable, it was evident that participants located risk in:

- the patient (e.g., ‘*the patient’s risk’*) which was sometimes qualified by the type of harm envisaged (e.g., ‘*his risk involved hurting staff’*);
- clinical activity (e.g., risk assessment, positive risk-taking, risk management);
- the clinician (e.g., risk tolerances and thresholds); and
- the system (e.g., ‘*our system is… quite risk averse*,’ ‘*who holds the risk’*).

**Image:**

**Figure:**
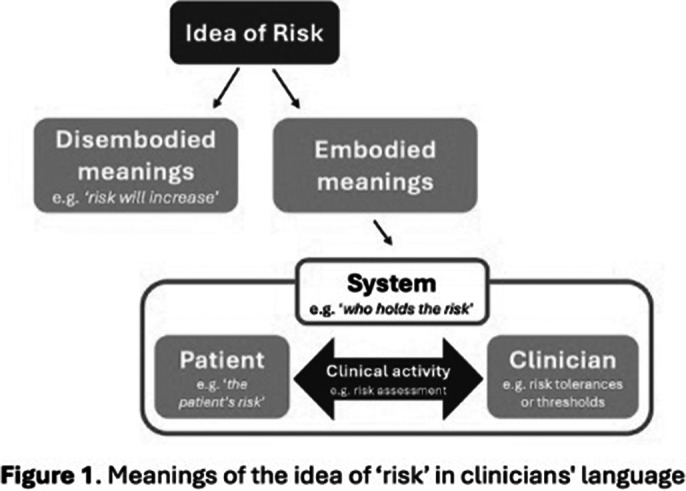

**Conclusions:**

This study demonstrates the varied use of ‘risk’ in practice. By empirically delineating the different expressed forms ‘risk’ takes in clinicians’ language (and thinking), the findings of this study can inform (i) the development of risk study methodologies that are more applicable to practice, and (ii) improvements in clinical practice by clarifying how risk can be understood and spoken about.

**Disclosure of Interest:**

None Declared

